# Screen-Printed Graphene/Carbon Electrodes on Paper Substrates as Impedance Sensors for Detection of Coronavirus in Nasopharyngeal Fluid Samples

**DOI:** 10.3390/diagnostics11061030

**Published:** 2021-06-03

**Authors:** Muhammad Ali Ehsan, Safyan Akram Khan, Abdul Rehman

**Affiliations:** 1 Center of Research Excellence in Nanotechnology (CENT), King Fahd University of Petroleum & Minerals, Dhahran 31261, Saudi Arabia; meali@kfupm.edu.sa (M.A.E.); safyan@kfupm.edu.sa (S.A.K.); 2 Department of Chemistry, King Fahd University of Petroleum and Minerals, Dhahran 31261, Saudi Arabia

**Keywords:** SARS-CoV-2, electrochemical sensors, Graphene/Carbon, COVID-19 antibodies

## Abstract

Severe acute respiratory syndrome (SARS-CoV-2), the causative agent of the global pandemic, which has resulted in more than one million deaths with tens of millions reported cases, requires a fast, accurate, and portable testing mechanism operable in the field environment. Electrochemical sensors, based on paper substrates with portable electrochemical devices, can prove an excellent alternative in mitigating the economic and public health effects of the disease. Herein, we present an impedance biosensor for the detection of the SARS-CoV-2 spike protein utilizing the IgG anti-SARS-CoV-2 spike antibody. This label-free platform utilizing screen-printed electrodes works on the principle of redox reaction impedance of a probe and can detect antigen spikes directly in nasopharyngeal fluid as well as virus samples collected in the universal transport medium (UTM). High conductivity graphene/carbon ink is used for this purpose so as to have a small background impedance that leads to a wider dynamic range of detection. Antibody immobilization onto the electrode surface was conducted through a chemical entity or a biological entity to see their effect; where a biological immobilization can enhance the antibody loading and thereby the sensitivity. In both cases, we were able to have a very low limit of quantification (i.e., 0.25 fg/mL), however, the linear range was 3 orders of magnitude wider for the biological entity-based immobilization. The specificity of the sensor was also tested against high concentrations of H1N1 flu antigens with no appreciable response. The most optimized sensors are used to identify negative and positive COVID-19 samples with great accuracy and precision.

## 1. Introduction

Severe acute respiratory syndrome coronavirus 2 (SARS-CoV-2) is a highly contagious virus, transmissible from human to human [1], which has been classified as a global pandemic by the World Health Organization [2]. Despite the rollout of many vaccine candidates in the last few months [3], much of the world population is still effected by outbreaks as new variants are identified and low-income communities have no access to vaccines [4]. Even where these vaccines are available, economic activities are still hindered, with schools and colleges most affected by apprehensions of outbreaks. Rapid, low-cost and accurate testing, particularly in the early days of infection [5] when the viral load is too small to easily cause false negatives, is of high significance [6,7,8]. Therefore, batch fabricated mass diagnostic devices or biosensors are needed among these communities to reduce the number of undetected cases [9]. Such an early and prompt diagnosis can play a crucial role for informed decisions concerning the isolation of infected patients. This, again, has an economic impact if a false positive person is isolated, while slowing the spread of this infectious disease by isolating the true positives [10,11,12].

After the genetic code of the virus was discovered, polymerase chain reaction (PCR)–based tests became the gold standard around the world [13,14]. However, these have their own limitations, the most important of which is a relatively long detection time that has been shortened considerably by many scientific developments in the last months, yet is still without a point-of-care possibility. Moreover, sample transport to specialized labs and the complicated sample pretreatment steps are a major hurdle to quick identification [11]. Such centralized diagnostic services provided by skilled personnel are not recommendable for rapid screening in public places and are only suitable for final verification. This has led to identification by body temperatures, which is not a true indicator on one hand, but more importantly is absent in the case of asymptomatic infections. Hence, a sensitive and inexpensive immunological tool is an essential part in our fight against the virus, preventing and controlling outbreaks, and re-opening the essential components of life [10].

Biosensors [15], with their large array of diagnostic applications and fast, easy, reliable detection, are a handy tool in this regard [16,17]. Several kinds of biosensors have been reported in the past for the diagnosis of different viruses. The mechanisms used for those detections include surface plasmon resonance [18], electrochemical transduction [19,20], including the impedance-based analyses [21,22], and colorimetric [23] lateral flow assays (LFAs) [24], which mostly target a specific antigen-antibody interaction. Some of these mechanisms have already been employed in biosensors [25] for the detection of SARS-CoV-2. Specific examples include a graphene-based field-effect transistor (FET) biosensor [26], a plasmonic photothermal biosensor [27], a fluorescence-based microfluidic immunoassay [28], and a small variety of electrochemical and impedance biosensors using commercial screen-printed electrodes. In one of these reports, the carbon electrodes were functionalized with the Cu_2_O nanocubes to enhance the surface area of the electrode for improving the functionality of the impedance-based mechanism [29]. The sensor explored the same COVID-19 spike protein-spike antibody interaction through another protein mediated immobilization of the antibody. In another effort, electrochemical immunosensing was explored, targeting the nucleocapsid protein and its complementary antigen of COVID-19 [30], where the authors utilized cotton swabs on the tip of the commercial electrode to improve direct sampling. A label-free, commercially available impedance sensing platform, using specialized well plates with integrated sensing electrodes from ACEA Biosciences, was also developed for the detection of COVID-19 antibodies [31]. A magnetic bead-based electrochemical assay, targeting spike protein and nucleocapsid protein using a labeled mechanism, was also demonstrated. This mechanism detected the enzymatic by-product using screen-printed electrodes modified with carbon black nanomaterial [22]. Another example, the cobalt-functionalized TiO_2_ nanotube sensor, was presented for the rapid detection of the virus through antibody-antigen interactions [32]. A paper-based approach is also demonstrated [12]. For this, the authors used well established EDC/NHS chemistry to graft the antigens of the graphene oxide electrodes and showed the detection of complementary antibodies, both IgG and IgM. These solitary examples of commercial screen-printed electrodes, in most cases, indicate that there is a wide gap in the fabrication protocols, the cost of testing, and the implementation of the resulting sensors. If some of these antigen-antibody interactions can be efficiently transferred onto paper or other low-cost substrates with the measurements done through portable devices, it would truly transform testing and isolation in the real world in real-time. 

With these objectives, we started to explore paper substrates for detection purposes. Among the many sensing mechanisms, electrochemical and impedance transduction is highly suitable due to its good sensitivity, low cost, low response time, and small sample requirement [22,29,31]. More importantly, the amenability to miniaturization, and subsequent portability, through already available devices provides it with immense potential for screening and POC testing. For the electrode materials, carbon and graphene nanostructures are considered ideally suited because of their large surface area, stability, and ease of functionalization [33]. In this study, the graphene-carbon electrodes were screen-printed onto cellulose based paper pad substrates in a batch fabrication mode using different masks. A commercially available ink with high conductivity was used for this purpose. Such high conductivity of electrodes (immobilizing matrix) in the presence of probe molecules (electron transport system) provide very low background signal, which results in high sensitivity of the measurement. The ease of functionalization in these graphene materials can also provide a flexibility in immobilizing antibodies to the electrode surface. Moreover, this whole arrangement provided a stable deposit of the electrode material which is required to bear the buffering conditions during the measurements. The selection of antibody immobilization method is another point of consideration in the fabrication as well as in the performance in its final form [34]. Some of the immobilization can be random and some can graft the antibodies in an oriented assembly. The defined orientation can enhance the antibody loading and interaction capacity, thereby increasing the functionality of the sensor [35,36]. Furthermore, the antibodies can be immobilized through chemical and biological mediators. We used two of these strategies to immobilize IgG antibodies of the SARS-Cov-2 spike protein and evaluated the performance of the paper-based sensors. For measurement purposes, we used portable devices from which the data was directly collected onto a mobile device via Bluetooth. Finally, the optimized sensors were used to screen through the nasopharyngeal fluid of the healthy and infected patients in order to compare with the results of PCR tests. 

## 2. Materials and Methods

### 2.1. Chemicals and Reagents

All chemicals and reagents used were of the highest purity available. Potassium ferricyanide (K_3_Fe(CN)_6_), potassium ferrocyanide (K_4_Fe(CN)_6_), staphylococcal protein A (ProtA), bovine serum albumin (BSA), and phosphate buffer saline (PBS) were obtained from Sigma-Aldrich (Germany). The 1-pyrenebutanoic acid succinimidyl ester (PBASE) was purchased from Fisher Scientific (Germany). SARS-CoV-2 spike protein (RBD) (cat no.: Z03483-100; Z03483-1) and SARS-CoV-2 spike S1 IgG antibody (cat no.: A02038-1) were purchased from GenScript Inc., (Piscataway, NJ, USA). Influenza A antigen (N1H1) (no. J8034) was purchased from BiosPacific, (Emeryville, CA, USA).

### 2.2. Fabrication of Sensing Electrodes

**Figure 1:**
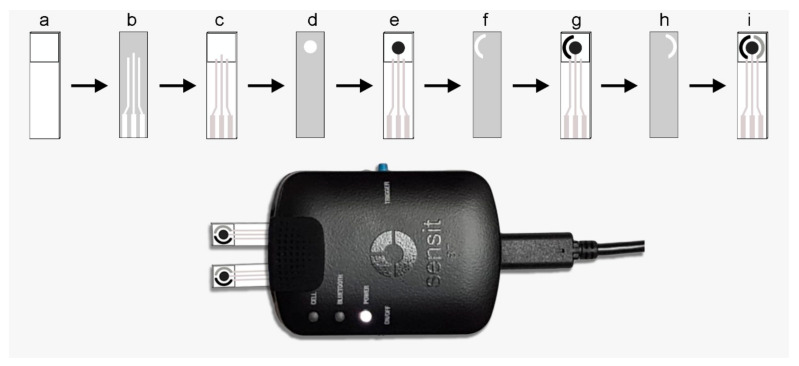
Schematic for the fabrication protocol of the paper-based impedance sensor and the measurement device with the sensors installed, where: (**a**) is the paper strip cut into appropriate size and paraffin-impregnated within defined test area; (**b**) is the mask 1 for the connecting wires and connectivity pads; (**c**) is the screen-printed connectivity pads and lead wires using the silver conductive paste; (**d**) is the mask 2 for the working electrode; (**e**) is the screen-printed working electrode using the high conductivity graphene/carbon hybrid ink; (**f**) is the mask 3 for the counter electrode; (**g**) is the screen-printed counter electrode with carbon ink; (**h**) is the mask 4 for the reference electrode; and (**i**) is the screen-printed reference electrode using the Ag/AgCl ink.

Single strip sensing electrodes were fabricated on cellulose fiber-based paper pads from Millipore, Germany (CFSP001700). For this purpose, the sensing strips were cut out of rolls into the appropriate size and dried in an oven at 140 °C. The design of the sensing area, as well as of the electrode structure, was made using Adobe Illustrator software. First, a pattern of the testing area was drawn by impregnation of paraffin film (Sigma-Aldrich (Germany), cat. No. 327204) using a hot metallic pattern and pressure transferring, resulting in a testing channel. A screen-printing and batch fabrication procedure was adopted to obtain the sensing devices as schematically outlined in Figure 1, involving different masks and inks. High conductivity graphene/carbon hybrid ink (Dycotec Materials, UK DM-GRA-9101S) was used to print the working electrode. Ag/AgCl ink (Creative Materials (Ayer, MA, USA) 119-10) was used to print the reference electrode. Carbon ink (Dycotec Materials, UK DM-CAP-4311S) was used to print the counter electrode. Between each printing of the electrodes, the strips were temperature cured at 100–140 °C for 30 min each. The connecting pads and the leading wires were also screen printed using conductive silver ink (Dycotec Materials, UK DM-SIP-3060S) and temperature cured. The non-exposed area of the strip was then covered with the UV-curable insulator ink (Dycotec Materials, UK DM-IN-7010S). After the UV-curing, these paper-based sensing strips were ready for the immobilization of biosensing elements. However, before the modifications, the reproducibility of the fabrication protocol was tested. In one batch, ten electrodes were fabricated, and repeatability and working of the testing area was examined by cyclic voltammetry in the presence of 5 mM K_3_(Fe(CN)_6_). Relative standard deviation (RSD) for the anodic peak current, calculated from these measurements, was less than 10% in each case. 

### 2.3. Immobilization of the Sensing Elements

For the direct immobilization of COVID-19 antibodies onto the electrode surface, 1-pyrenebutanoic acid succinimidyl ester (PBASE), an interface coupling agent, was used as a probe linker. For this purpose, the fabricated paper electrode was impregnated with 10 µL of PBASE (10 mg/mL) in methanol for 1 h at room temperature. After washing with PBS, the functionalized sensor was then incubated with 10 μL of IgG solution (30 mg/mL) in PBS (pH 7.4) for 1 h. The free sites on the electrode surface, which otherwise can cause non-specific interactions, were blocked by a solution of 0.1% BSA in the PBS buffer. In another attempt, a more oriented immobilization of spike S1 IgG antibody was achieved, using 10 μL of ProtA solution (10 mg/mL) in PBS (pH 7.4), impregnated onto the surface of the graphene electrode for 1 h. The IgG antibody was then immobilized by incubating it with 10 μL of its solution (30 mg/mL) in PBS for 0.5 h. Again, the active sites were blocked from the unspecific interactions by 10 μL of BSA (0.1%) solution dropping onto the modified electrode and washing with PBS. For both cases of direct immobilization of antibodies and the ProtA-mediated one, the test strips were then stored in dry conditions at 4 °C in a refrigerator until immediately before use. A check of the surface, after the modification was done, was performed through SEM analysis. However, this analysis was only intended to look into the orientation status of the immobilization. For that purpose, a field-emission SEM instrument (Lyra 3, Tescan, Czech Republic) was used. 

### 2.4. Electrochemical Measurements

All the electrochemical data was collected using a battery-powered portable potentiostat/impedance analyzer (PalmSens4, PalmSens BV, The Netherlands) transferred to a laptop and, in some cases, to a mobile device using Bluetooth connectivity. In order to demonstrate the portability of the sensing devices, the optimized sensing electrodes were also validated using SensIT BT devices (PalmSens BV, The Netherlands). Cyclic voltammetry (CV) and electrochemical impedance spectroscopy (EIS) were used to study the different steps of immobilization, whereas the quantitative measurements were done using EIS only. For each measurement, the required volume of the test solution was impregnated onto the electrode surface at room temperature and the data was collected after a fixed interval of time. For each exposure, the given sample quantity was dropped onto the sensor surface and the sensor response was obtained after 5 min. This time duration was kept constant throughout the study for all the experiments.

### 2.5. Treatment of the Samples

Both types of antibody modified strips were tested against the RBD antigen in artificial sample matrices, i.e., nasal fluid from a healthy volunteer collected in PCR tubes spiked with antigen solutions. For the analysis of real samples, four nasopharyngeal swab samples were collected from a local hospital, which were identified to be positive (three cases) and negative (two cases) by RT-PCR. These samples were collected in the universal transport media (UTM), inactivated by heating at 100 °C for 10 min, and stored in the freezer at −80 °C until tested with the fabricated strips. All the real sample testing was done using the portable devices in a bio-safe environment, whereas the analytical team was blinded to clinical information of the patients and the samples. 

## 3. Results and Discussion

### 3.1. Sensor Fabrication and Characterization

**Figure 2:**
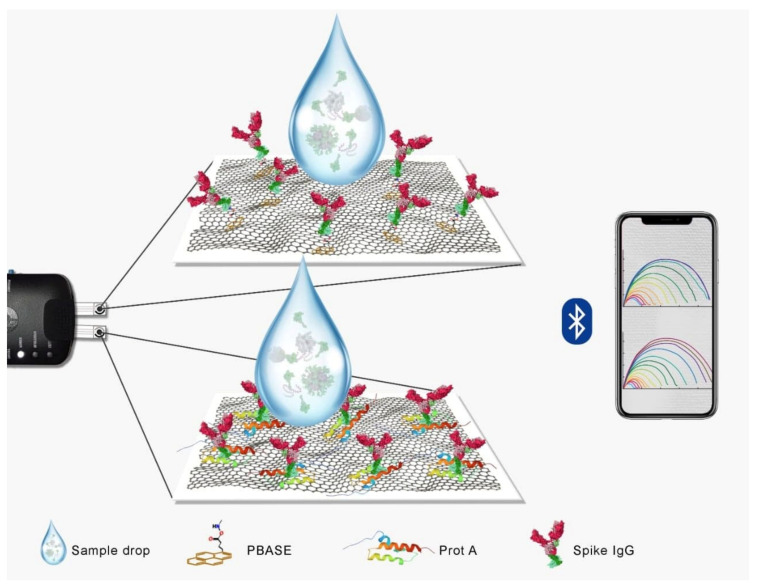
A schematic illustration of the strategies adopted in this work for immunosensing of the coronavirus in the nasopharyngeal samples using paper substrates, and graphene electrode. The difference between the two strategies is also shown where the immobilization in the top of the frame is PBASE mediated one and the immobilization in the bottom frame is protein mediated one. The sensor signals were transmitted to a mobile device using Bluetooth.

SARS-CoV-2 spike S1 IgG antibody, as a biosensing element, is required to have a strong immobilization on the electrode surface, with a good homogenous orientation, in order for the sensor to work efficiently towards COVID-19 detection. Moreover, the electrode material, i.e., graphene in this case, must be stable enough to bear the buffering conditions during different electrochemical processes. For impedance-based sensors, as proposed in this study, the conductivity of the sensing electrode needed to be high so as to extend the detection range by providing a sharper and low baseline impedance. In order to achieve that synchronized situation, high conductivity graphene hybrid ink (Sheet Resistance = 10 Ω/Sq/25 µm) was used and two different strategies were applied and tested for their final outcome. In the first case, (PBASE), an interface coupling agent typically used as a probe linker that binds to IgG, was immobilized on the hydrophilic cellulose paper pads. Graphene/carbon film was tightly embedded in the porous cellulose network in the working zone of the sensor due to the presence of hydroxyl groups both on paper and graphene. Physisorption, as well as the hydrogen bonding in the two structures, can lead to an integrated structure in such a scenario, thereby avoiding a layer of film peeling off once the surface is rehydrated by the buffer systems. Thus, the stability and reproducibility of the fabricated sensor can be significantly enhanced. The activated carboxylic terminals of the graphene can then be used to incorporate PBASE and, later, for the attachment of IgG antibodies. Such interaction can also occur between the graphene and pyrene groups of the PBASE to support this immobilization mechanism. At the same time, a protein-mediated IgG immobilization strategy using the same paper substrate was also applied. ProtA, a strong immunological instrument, was used in this regard, which can strongly bind to the Fc region of IgG antibodies, leading to well-defined orientations of the antibody elements. In an earlier report [29], this strategy was tested on Cu_2_O modified commercial electrodes with good recognition ability for the spike proteins. In this study, we utilized graphene on paper electrodes using the same strategy. The immobilized antibodies with such defined orientation were proven to increase the antigen-binding capacity of the films, thus improving the function of the detection system. A schematic illustration of the two strategies is provided in Figure 2. 

The electrochemical profile at each fabrication step was studied by two complementary techniques (i.e., EIS and CV) in ferro/ferricyanide solution. The EIS is regarded as a method that can obtain electrical information in a broad frequency range in order to monitor the electrode modification steps. Herein, the EIS data was fitted into Randles’ equivalent circuit model, whereas the real and imaginary components of the impedance were plotted as the Nyquist plot. The straight diagonal line in the Nyquist plot at lower frequencies indicates a typical behavior of the planar electrodes for diffusion-controlled redox reactions, while the semicircle part at higher frequencies represents the electron transfer efficiency between the redox couple and the electrode surface, which is known as charge transfer resistance (Rct). The Rct values were used as the sensor signal for different measurements. CV, on the other hand, provides indications in the form of oxidation and reduction peak heights that are increased or decreased based on the changes of the charge transfer kinetics. 

**Figure 3:**
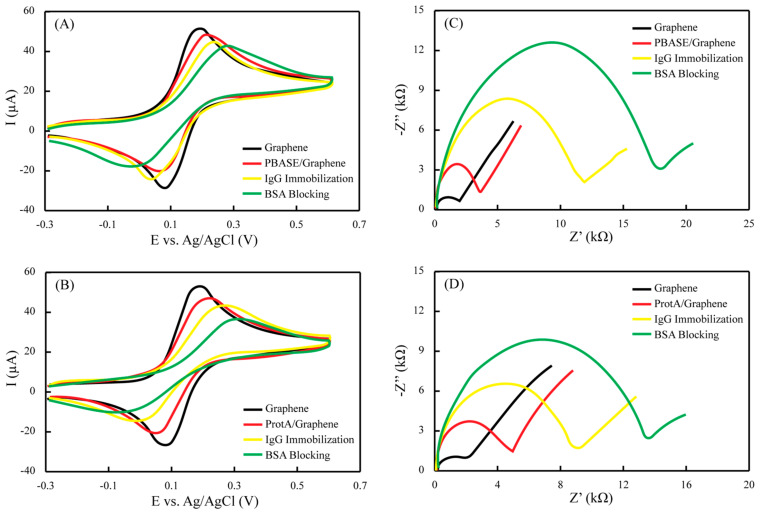
CV and EIS characterization of the immobilization protocols for paper-based immunochemical sensor, where (**A**,**B**) represent the changes in the redox processes at the interface and the shifts of the redox potentials with the addition of different biochemically interacting species, and (**C**,**D**) represents the changes in the impedance of the electrode surface due to the introduction of more insulating species during immobilization.

Figure 3 illustrates the state for each step of the sensor fabrication in terms of both CV and EIS. Starting with two different electrodes, the peak currents and Rct were measured simultaneously for PBASE and ProtA-mediated binding. Both the electrodes chosen have almost the same values for the oxidation peak current (~50 µA) and Rct (~2 kΩ). The peak potentials for the reduction and oxidation are sufficiently close to indicate the reversible reaction and a minimal obstruction towards the redox conversion. When the graphene sensor surfaces were modified with PBASE, which is a molecular entity, the redox peak current, as well as the Rct, only slightly shifted for the ferro/ferricyanide couple (Figure 3A,C). On the other hand, when a similar electrode was modified with ProtA, which is a biological entity of larger size domain and thus causing a more insulating behavior, the redox peaks, as well as the Rct, was shifted to a larger extent (Figure 3B,D). These observations suggest that the added reagents behaved as insulators, thereby impeding the electron transfer at the interface. However, this behavior was somewhat reversed in the case of antibody immobilization. Overall, the continuous enlargement of the Rct was still observed, implying that the immobilization of the spike protein was accomplished. However, for the antibody immobilization over the PBASE, which is considered a relatively random process, the Rct shift was significantly higher as compared to ProtA-mediated binding, which is a relatively organized and oriented process. Similar voltammetric and impedimetric behavior was observed when the non-active sites were blocked with BSA in both cases, thereby leading to the sensor surfaces having an appreciably lower impedance baseline to conduct further quantitative tests. 

### 3.2. Quantitative Detection of RBD in Nasopharyngeal Samples

**Figure 4:**
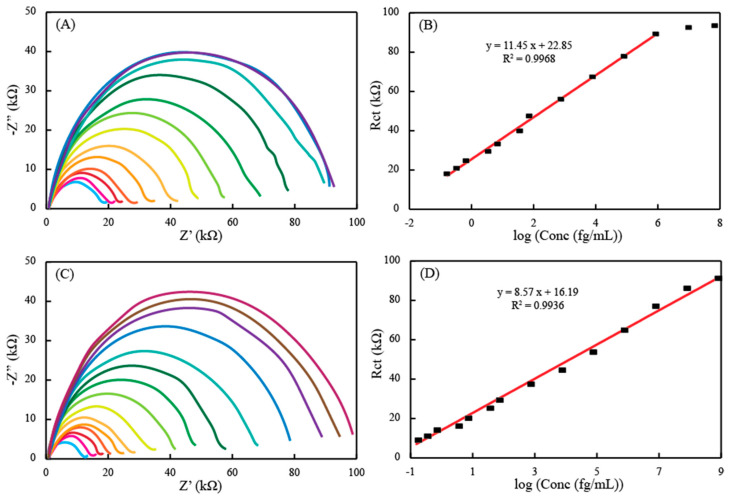
The sensor response of different concentrations of the RBD spiked directly in diluted nasopharyngeal samples and the corresponding calibration plots, obtained with the linear regression equation shown here, (**A**) represents the concentrations in the range of 0.25 fg/mL to 100 ng/mL measured using the PBASE-mediated antibody immobilized electrode on graphene with (**B**) as its calibration plot and (**C**) represents the concentrations in the range of 0.25 fg/mL to 1 µg/mL measured using the ProtA-mediated antibody immobilized electrode on graphene with (**D**) as its calibration plot.

The performance of the sensor thus fabricated was quantified against a series of concentrations of RBD antigen (spike protein) using EIS. In order to have more practical applicability, a nasopharyngeal sample of a healthy donor was obtained and diluted. The aliquots of this sample was spiked with different concentrations of the antigen. Figure 4A indicates the EIS responses for the increasing concentrations of the RBD in the nasopharyngeal samples when the antibody-immobilized sensor, through PBASE mechanism, was exposed to these concentrations ranging from 0.25 fg/mL to 1 × 10^8^ fg/mL. A gradual increase in the Rct semicircle in the Nyquist plots was observed with the increasing concentration of the spike protein. This indicates the inhibition effects of the RBD for the electron transfer between the electrode surface and the redox couple. Even small changes in the concentrations can lead to the altered interfacial processes, demonstrating the specific binding of the protein with the antibodies immobilized on the surface and also providing an idea of the sensitivity of the sensor probe. The response of the sensor (Rct) was plotted against the log of the concentrations spiked in the nasopharyngeal samples (Figure 4B). This relationship was found to be linear in the range of 0.25 fg/mL to 1 ng/mL with a regression equation of Rct = 11.45 log C + 22.85. After this linear range, the saturation level of the sensor was reached, and so, there was no increase in the Rct values with the increase of the concentration of RBD. Still, the concentration range and the limit of detection was better than typically available ELISA platforms, which can be attributed to the high conductivity of the graphene electrodes fabricated in this study. A similar set of experiments were performed with the sensor strips modified with antibodies through the ProtA mechanism, which are supposed to have a much more aligned antibody structure. The data is provided in Figure 4C. The response obtained from the spiked nasopharyngeal samples was also similar to the PBASE-modified antibody sensor, i.e., the EIS impedance increased with the increasing concentration of the RBD and that response was linearly proportional to the log of the concentration. However, the linear range was extended 3-fold in magnitude of the concentration (i.e., up to 1 µg/mL) for the antigen in the samples, as shown in Figure 4D. As the electrode surface was the same graphene, this enhancement of the sensor signal can be attributed to the ordered orientation of the antibody through ProtA-modification. Despite of this wider dynamic range of measurement, the limit of quantification has more of a significance here. This is due to the fact that the sensors have to be utilized in the detection of real samples invaded by the virus itself. The virus loading in the patient’s biological fluids exponentially increase based on the number of days that have passed after infection. This phenomenon can lead to false negatives in the early days after infection if the detection mechanisms have higher limits of quantification, as the virus load is very low from day 1 to day 4 of the infection. This is the time when the patient can be identified as infected and thereby isolated, and further infections can be limited as the patient is relatively less contagious in this timeframe. Both the EIS sensors presented here are capable of quantifying 0.25 fg/mL of the concentration, which is sufficiently low enough to enable early identification and isolation of the infected person. However, this number depends on many factors, such as the number of days the patient has been infected or even the inherent immunity of the patient themself. 

When compared to the performance of this sensor against recently published sensor reports, the sensitivity was comparable to an FET sensor (1 fg/mL), a monoclonal antibody sensor (0.1 µg/mL), an electrochemical sensor based on Cu_2_O nanocubes (0.25 fg/mL–1 µg/mL), an electrochemical sensor based on gold nanoparticles (1 pg/mL), a cotton tipped electrochemical sensor (0.8 pg/mL), a molecularly imprinted electrochemical sensor (50 fM), and a plasmonic photothermal biosensor (0.22 pM). However, the condition of measurement and sampling were different in all the cases, therefore, a true comparison is neither possible nor justifiable.

**Figure 5:**
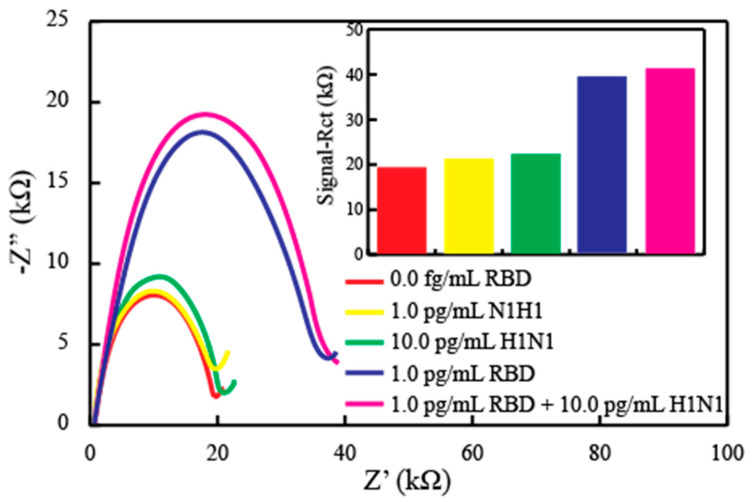
The fabricated sensor exposed to different concentrations of the specifically interacting antigen (RBD) and non-specific antigen (H1N1), in solitary and mixture format, to allow the selectivity measurements of the sensor. The inset shows the bar graph of the responses obtained.

Furthermore, the sensor can only have a practical value if the detection mechanism is selective, which is rather specific in the case of such critical detections where false positives mean a person is isolated from most of his/her activities for a period of at least two weeks. To confirm this selectivity, the oriented antibody sensor was exposed to potential interfering agents. In order to confirm the behavior of the sensor, a fabricated strip was subjected to EIS measurements against the Flu A antigen up to 10 pg/mL of the concentration, again in nasopharyngeal samples. Figure 5 shows the EIS curves with an inset showing a bar graph of the responses. For the antigen H1N1, the sensor showed a negligible increase in the impedance as there would be no binding of this antigen on antibody sites. After PBS washing, the same sensor was exposed to 1.0 pg of the RBD, and the sensor showed significant signal to indicate again the specific binding. This binding remains intact even if the sensor is exposed to the mixture of the RBD with 10 times the concentration of the possible interferent. These results indicate high selectivity of the proposed EIS immunosensors.

### 3.3. Real Sample Testing

**Figure 6:**
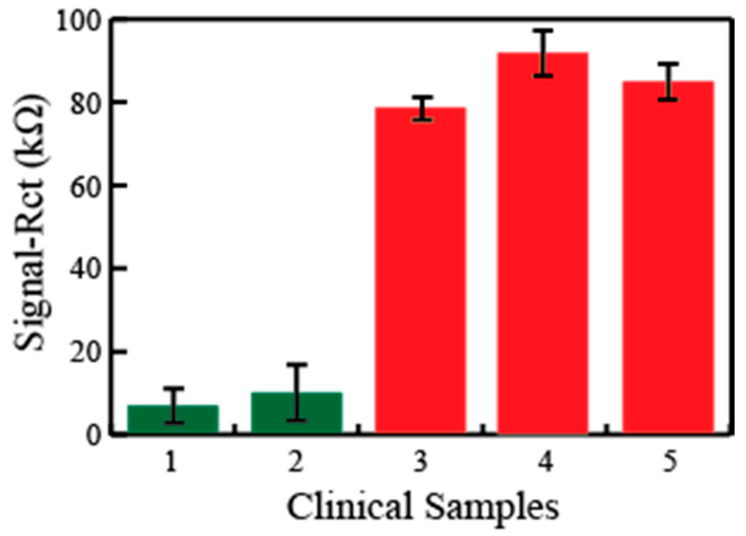
The testing of five clinical samples taken as three aliquots in each case and measuring the impedance on the sensor surface. The green bars indicate negative and red bars indicate the positive detection of the COVID-19 viral disease.

Next, we investigated the EIS sensor’s ability to detect the SARS-Cov-2 virus in real samples. To this end, the nasopharyngeal swab specimens from including COVID-19-negative (*n* = 2) and COVID-19-positive samples (*n* = 3), already confirmed by gold standard PCR tests, were collected and stored in UTM. Each sample was divided into three aliquots in order to study the reproducibility of the data as well. When the disposable sensor strips were exposed to these sample aliquots, a clearly distinguishable response was observed in the case of both the negative and positive samples (where 1 and 2 represent the negative samples and 3–4 represent the positive samples) in Figure 6. Further, these responses are quite reproducible when different aliquots of the same sample were used as shown by the standard deviation values. This exhibits a great promise for a reliable detection platform for the fast indication of the disease directly in the real samples.

## 4. Conclusions

In summary, we herein presented a low-cost, batch fabrication protocol for a cellulose paper substrate, where the highly conductive formulation of the graphene/carbon ink can be screen-printed as a working electrode to be immobilized with the antibodies in different formats. A PBASE directed format and a ProtA controlled format were tested, which were characterized by the electrochemical techniques. Both the tested formats generated appreciable EIS signals as the viral antigen (RBD) or the virus itself when attached, impeding the redox reaction of the (Fe(CN)_6_)^3−/4−^. The sensors could quantify the concentrations with as little as 0.25 fg/mL in spiked nasopharyngeal samples. Moreover, the sensor showed high selectivity and reproducibility. The ProtA antibody immobilization resulted in a wider dynamic range. Therefore, this sensor was further utilized in the real samples to indicate the positive and negative COVID-19 identity in the samples with a correct identification as confirmed by standard PCR testing. It is important to note that the sensor data was collected using portable potentiostats, where the signals are transferred to smart devices via Bluetooth. Thus, it can be concluded here that the fabricated sensors show promise in direct, rapid, and low-cost diagnosis without sample pretreatments. Moreover, the sensor fabrication process can be automated, and such developments are underway in our lab. With the development of more stable and high affinity receptors, natural (monoclonal antibodies) or synthetic (artificial antibodies based on polymers), such sensors have the potential to be implemented in the day-to-day screening of COVID-19. 

## Data Availability

Data is contained within the article.
